# Deciphering the Genomic Characterization of the GGP Gene Family and Expression Verification of *CmGGP1* Modulating Ascorbic Acid Biosynthesis in Melon Plants

**DOI:** 10.3390/antiox13040397

**Published:** 2024-03-26

**Authors:** Tiantian Yang, Sikandar Amanullah, Shenglong Li, Peng Gao, Junyu Bai, Chang Li, Jie Ma, Feishi Luan, Xuezheng Wang

**Affiliations:** 1 College of Horticulture and Landscape Architecture, Northeast Agricultural University, Harbin 150030, China; yttneau2021@gmail.com (T.Y.); amahanekuroha@gmail.com (S.L.); gaopeng_neau@163.com (P.G.); baijunyu11@126.com (J.B.); s220401055@neau.edu.cn (C.L.); luanfeishi@neau.edu.cn (F.L.); 2 Key Laboratory of Biology and Genetic Improvement of Horticulture Crops (Northeast Region), Ministry of Agriculture and Rural Affairs, Harbin 150030, China; 3 Bayannur Institute of Agriculture and Animal Husbandry Science, Inner Mongolia Autonomous Region, Bayannur 015000, China; nmgmj163@163.com

**Keywords:** acclimatization, *Cucumis melo* L., germplasm, L-galactose pathway, metabolism

## Abstract

Ascorbic acid (AsA), also known as vitamin C, is a well-known antioxidant found in living entities that plays an essential role in growth and development, as well as in defensive mechanisms. GDP-L-galactose phosphorylase (GGP) is a candidate gene regulating AsA biosynthesis at the translational and transcriptional levels in plants. In the current study, we conducted genome-wide bioinformatic analysis and pinpointed a single AsA synthesis rate-limiting enzyme gene in melon (*CmGGP1*). The protein prediction analysis depicted that the CmGGP1 protein does not have a signaling peptide or transmembrane structure and mainly functions in the chloroplast or nucleus. The constructed phylogenetic tree analysis in multispecies showed that the CmGGP1 protein has a highly conserved motif in cucurbit crops. The structural variation analysis of the *CmGGP1* gene in different domesticated melon germplasms showed a single non-synonymous type-base mutation and indicated that this gene was selected by domestication during evolution. Wild-type (WT) and landrace (LDR) germplasms of melon depicted close relationships to each other, and improved-type (IMP) varieties showed modern domestication selection. The endogenous quantification of AsA content in both the young and old leaves of nine melon varieties exhibited the major differentiations for AsA synthesis and metabolism. The real-time quantitative polymerase chain reaction (qRT-PCR) analysis of gene co-expression showed that AsA biosynthesis in leaves was greater than AsA metabolic consumption, and four putative interactive genes (*MELO3C025552.2*, *MELO3C007440.2*, *MELO3C023324.2*, and *MELO3C018576.2*) associated with the *CmGGP1* gene were revealed. Meanwhile, the *CmGGP1* gene expression pattern was noticed to be up-regulated to varying degrees in different acclimated melons. We believe that the obtained results would provide useful insights for an in-depth genetic understanding of the AsA biosynthesis mechanism, aimed at the development of improving crop plants for melon.

## 1. Introduction

AsA is an important trace element that is required for the primary functions of the typical growth and development of plants and the human body against adverse stress conditions [1]. In plants, it exists in a varied array of tissues and serves as a main redox buffer. It is a versatile metabolite associated with several biological activities, e.g., the regulation of photosynthesis activity, cell wall biogenesis, regulation of seed germination, influencing flowering time, hormone biosynthesis, fruit maturation and softening, and generating new antioxidants facilitating signal transduction and boosting plants resistance in biotic and abiotic stress phases [1,2,3,4,5,6,7,8].

Endogenous biosynthesis is the primary factor affecting the molecular regulation of AsA levels within an organism and is influenced by normal biosynthetic and metabolic pathways [1,3,9]. AsA mainly acts as a powerful antioxidant, neutralizing the free radicals generated as a result of regular metabolic processes or in reaction to stress, thus preventing damage to the cells in plants [10]. The rapidly developing plant tissues exhibited the highest activity of ascorbate oxidase, regardless of whether they are from fruits or leaves [8], and this has the potential to regulate a variety of signaling pathways [11]. The natural synthesis of AsA mainly yields L-type and D-type structures. D-AsA itself is not biologically active and L-AsA mainly functions as an antioxidant [12]; however, the chemical structure of AsA is extremely unstable and easily loses its activity due to oxygen, light, high temperatures, and alkaline substances. AsA deficiency leading to elevation in the reactive oxygen species (ROS) levels within the cell compartments, destroying the protoplasmic membrane structure and causing a decrease in the active transport ability of cells, eventually leading to the expulsion of intracellular proteins and other substances, as well as causing irreversible damage to cells in severe cases [13]. In particular, the human body cannot synthesize AsA by itself and a lack of AsA is likely to cause major symptoms such as bleeding gums, joint pain, rapid aging, cancer, and further serious diseases [14,15]. Human beings need to rely on fresh and healthy plant food to obtain certain AsAs [16]; hence, it holds significant importance to study AsA synthesis in targeted crop plants.

The complete elucidation of the pathway contributions to AsA synthesis is still incomplete in many plants. At present, there are four possible suggested pathways, as follows: the L-gulose pathway, the L-galactose pathway, the D-galacturonic acid pathway, and the inositol pathway, which are well known for AsA biosynthesis. Of these, L-galactose is thought to be the main pathway regulating AsA synthesis in higher plants [12,17]. This pathway mainly contains five important enzymes that perform mutual biological and catalytic functions for AsA synthesis from GDP-D-mannose in the following different steps: (1) GDP-D-mannose 3′,5′-epimerase (GME) changes the GDP-D-mannose to GDP-L-galactose 1-phosphate [18,19]; (2) the conversion of GDP-L-galactose phosphorylase (GGP) into L-galactose phosphorylase [20,21]; (3) L-galactose-1-phosphate phosphatase (GPP) transforms the L-galactose-1-phosphate into L-galactose [22,23]; (4) L-galactose dehydrogenase (GalDH) changes L-galactose to L-glactose 1,4-lactone [12,24]; and (5) L-Galactono-1,4-lactone dehydrogenase (GalLDH) converts the L-Glactose-1,4-lactone into AsA [25,26].

GGP, identified as the rate-limiting enzyme, serves as the primary step involved in the pathway of L-galactose biosynthesis, exerting a significant impact on the AsA synthesis in numerous crop plants [27]. The main role of GGP remained unknown until 2007 and the gene encoding GGP was the final gene to be cloned from the L-galactose pathway [28]. Furthermore, two candidate GGP genes (*VTC2* and *VTC5*) were discovered in Arabidopsis (*Arabidopsis thaliana*) [21]. The characterization, expression analysis, and functional regulation mechanisms of GGP genes have been stated in different crop species, e.g., *Arabidopsis thaliana* [20], *Arabidopsis thaliana* and kiwi (*Actinidia deliciosa*) [29], potato (*Solanum tuberosum*) [30], strawberry (*Fragaria* × *ananassa*) [31], blueberry (*Vaccinium myrtillus*) [32], and tomato (*Solanum lycopersicum*) [4,33]. It was also proved that GGP is a key regulatory enzyme, which triggers the internal biosynthesis of ascorbic acid levels and seems to increase in tobacco (*Nicotiana tabacum*) [34], rice (*Oryza sativa*) [35], bread wheat (*Triticum aestivum*) [36], and kiwi (*Actinidia deliciosa*) [37]. The molecular regulation in *Arabidopsis thaliana* mainly showed that the GGP gene was significantly up-regulated by more than 20 times and AsA content was also increased after 24 h of intense light treatment in leaves [20]. Light can perform a key role in triggering GGP gene expression [38,39]. The activity of major enzymes in the synthetic pathway was significantly up-regulated by twice the activity of the GGP enzyme, and other enzymes were not changed under the induction of strong light, which confirmed that GGP was a key regulatory gene in the L-galactose pathway synthesis of AsA under light treatment [40]. *AceGGP3*, a potential gene involved in AsA synthesis, was investigated in kiwifruit with significant AsA content differences. It was also found that the interaction of two genes (*AceMYBS1* and *AceGBF3*) mainly promoted the expression of the *AceGGP3* gene, triggering a significant upsurge in the AsA content in kiwi [37].

Melon (*Cucumis melo* L.) is a popular fruit in the Cucurbitaceae family, which is mainly classified into two subspecies (ssp. *melo* and ssp. *agrestis*) [41]. Based on the genetic diversities and identification of the chromosomal localization of candidate genes for breeding [42], melon germplasms have been categorized into improved variety (IMP), landrace (LDR), and wild type (WT) germplasms, depicting a broad divergence in morphology and quality traits [43]. Due to its broad genomic assets, melon has become an excellent model plant for dissecting the essential biological pathways involved in the regulation of numerous complex traits [44]. In recent years, due to the essential roles of AsA in enduring plant life activities, there has been a significant focus on exploring the AsA biosynthesis pathways and associated genes, particularly *GGP*. Genome-wide analyses for identification and characterization of the GGP gene family have been well-documented in wheat [36], leek [45], and citrus [46]. Although key genes governing the AsA biosynthesis in plants have been pinpointed, the understanding of the molecular and biological mechanisms by which GGP gene family members contribute to AsA biosynthesis is limited in melon.

Herein, we performed a comprehensive bioinformatics analysis for the genome-wide identification and characterization of the GGP gene family in melon. Further, the endogenous determination of AsA synthesis and the associated gene expression patterns were checked in the plant leaves of four melon germplasm resources (including nine varieties) during the plant growth activity. We believe that the identified results gave us an important genetic regulatory basis for figuring out how the GGP gene family works for AsA biosynthesis in melon.

## 2. Materials and Methods

### 2.1. Genome-Wide Bioinformatics Analysis for Identification of GGP Gene

First of all, the primary data (the GFF, CDS, and Pep files) of GGP gene family members was obtained by searching the reference genome of melon_DHL92_V4 publicly available on the Cucurbitaceae website (http://cucurbitgenomics.org/, accessed on 5 March 2023). The relevant dataset was downloaded and the targeted GGP gene was identified. Then, the GGP protein sequences were identified by searching on the online website of the National Centre for Biotechnology Information (NCBI) (https://www.ncbi.nlm.nih.gov/, accessed on 5 March 2023) and the obtained homologous sequences were aligned using the online BLAST search and multiple sequence alignment tool. Later, the primarily identified *GGP* genes obtained using the above two methods were combined and filtered to remove the duplicates.

The protein secondary structure analysis was performed based on the online Self-Optimized Prediction Method With Alignment (SOPMA) (http://npsa-pbil.ibcp.fr/cgi-bin/npsa_automat.pl?page=npsa_sopma.html, accessed on 15 March 2023). The protein tertiary structure analysis was performed using the online structural bioinformatics web server SWISS-MODEL (https://www.swissmodel.expasy.org/, accessed on 15 March 2023) and the predicted local distance difference test score (pLDDT, >70) method of the AlphaFold database (version 2.0). The protein signal peptides were predicted using the online SignalP server (version 5.0) and the transmembrane helices of integral membrane proteins structures were analyzed using the online server of TransMembrane prediction using Hidden Markov Models (TMHMM, version 6.0) (https://services.healthtech.dtu.dk/service.php?DeepTMHMM, accessed on 15 March 2023).

Then, the subcellular location of proteins was detected by operating the online tool “Plant-mPLoc” (http://www.csbio.sjtu.edu.cn/bioinf/plant-multi/, accessed on 25 March 2023). The NCBI function module “Cn3D macromolecular structure viewer” was utilized to display the binding positions of the functional domain. The protein–protein interaction (PPI) network of melon GGP was explored using the online STRING database (https://string-db.org/, accessed on 25 March 2023). Then, the downloaded data of the melon_DHL92_V4 genome (http://cucurbitgenomics.org/, accessed on 20 March 2023) was further used for GGP gene and its interactions analysis in different tissues of the melon plant, using the transcriptome sequencing data with BioProject ID (PRJNA383830) [47].

### 2.2. Genomic Evolutionary Relationship Analysis of GGP Protein Sequences

The GGP protein sequences were searched among different crop families (Cruciferae, Actinidiaceae, Cucurbitaceae, and Acaridae) using the online web database of NCBI (https://www.ncbi.nlm.nih.gov/, accessed on 12 April 2023). The homologous evolutionary tree for all GGP protein sequences was constructed with MEGA software (version 7.0) [48] using the proximity method (NJ) and 1000 bootstrap repetitions. The GGP genes of cucurbit crops were selected from a filtered bioinformatics dataset and the obtained complete gene structure and chromosomal information of GGP gene, comprising length of gene, CDS location, as well as prediction of function domain, was displayed using the Gene Structure View function of integrative toolkit of the Tbtools (version 2) software package.

Afterward, the conserved motifs of the melon GGP protein were identified based on the online database of MEME (http://meme-suite.org/, accessed on 23 April 2023). The motif length was fixed to 6 to 200 amino acids (aa), the number of main motifs was fixed to 10, and the final data were then saved. The multiple protein sequences of the GGP gene of cucurbit crops were aligned using the MEGA software (version 7.0) [48] and the results of comparative amino acid sequences were demonstrated through the Conserved Domain Database (CDD) function domain [49]. The domain site was analyzed by using the function domain binding site information on the NCBI website (NCBI-CDD) (https://www.ncbi.nlm.nih.gov/Structure/cdd/wrpsb.cgi, accessed on 23 April 2023).

### 2.3. GGP Gene Structure Analysis among Different Germplasm Resources of Melon

**Figure 1:**
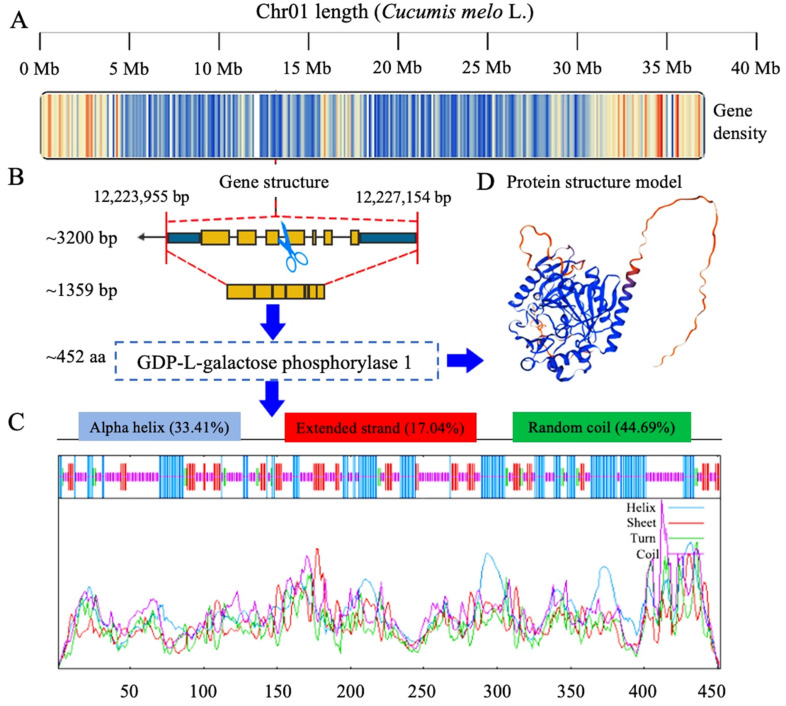
Genome-wide identification and characterization of *CmGGP1* gene in melon. (**A**) Gene density across the genetic length of Chr01. The color gradients indicate the gene density across the genetic length of the chromosome. (**B**) *CmGGP1* gene structure. (**C**) Prediction of CmGGP1 protein and membrane structure. (**D**) Prediction of CmGGP1 protein structure model. bp, base pairs; aa, amino acids.

A total of four types of melon germplasm resources “wild-type (WT, three varieties named PI 614174, PI 614410, and PI 614414), improved-type (IMP, two varieties named PMR45 and 16H), landrace-type (LDR, three varieties named Cinderella melon, PI 179914, and Shu Shu melon), not defined-type (ND, one variety named Xin Yin No.2)” belonging to the two subspecies (*Cucumis melo* ssp. *melo* and ssp. *agrestis*) were selected as experimental material (see Supplementary Table S1). The different geographical origins of the germplasm resources were visualized by drawing a worldwide map (Figure 1) using the R language tool (version 4.02). Whole genome DNA resequencing of all germplasm materials was downloaded from the NCBI GenBank (https://www.ncbi.nlm.nih.gov/, accessed on 28 April 2023), along with the BioProject (ID: PRJNA529037) and varieties of corresponding sample numbers [43].

The SNP-associated variant call format (VCF) file of resequenced accessions was obtained using the following methods: (1) clean end sequence reads were plotted on to the melon reference genome and SAM files were obtained using Burrows Wheeler Alignment software (v0.7.15-r1140) and (2) the obtained SAM file was converted into BAM and its indexed files [50]. The filtered VCF files of nine melon varieties were aligned in pairwise form and principal component analysis (PCA) was performed. The *CmGGP1* gene was searched in the comparative whole genome sequences and reference genomes of melon_DHL92_V4 (along with annotation) by operating the Integrative Genomic Viewer (IGV, version 2.4.4) software. Further, the multiple sequence differences were visualized using DNAMAN (version 9.0) by selecting the function of Align by Muscle command.

### 2.4. Endogenous Determination of AsA Content and Gene Expression Patterns

The seeds of nine melon varieties (PI 614174, PI 614410, PI 614414, PMR45, 16H, Cinderella melon, PI 179914, and Shu Shu melon) were cultivated in a big plastic greenhouse located at Xiangyang Experimental and Agricultural Base (45°07′ N, 125°430′ E), Harbin. A total of five plants from each melon variety were grown in a completely randomized design (CRD), following three replications, and Plant × Plant (65 cm) and Row × Row (45 cm) distance were maintained. All the integrated cultural practices were applied to attain the better development of crop plants.

Regarding the endogenous determination of AsA content (mg/100 g), a total of 5 g of young leaves (true leaf stage) and 5 g of old leaves (fully expanded leaf stage) were freshly sampled after being weighed from the replicated plants of each melon variety and were quickly stored in ultra-freezing liquid nitrogen, respectively. The endogenous AsA content in the leaves was quantified through a method of catalytic titration with hexavalent chromium by using a single-beam UV–Vis spectrophotometer (model CECIL 121, England), as reported by Abera et al. [51].

The gene expression pattern was determined by performing an analysis of the quantitative real-time polymerase chain reaction (qRT-PCR). In short, the fresh samples were collected from respective young true leaves and fully expanded old leaves, and a good-quality total RNA was isolated using the Trizol reagent protocol, as earlier reported by Rio et al. [52]. The purified first-strand complementary DNA (cDNA) was synthesized using the PrimeScript RT Master Mix Perfect Real-Time kit (Toyobo, Osaka, Japan). The primers of the *CmGGp1* gene and interactive genes were exported through Primer Premier software (version 6.25) [53] and *Actin*7 was used as a reference gene, as earlier reported [43]. All the gene primer information can be seen in Supplementary Table S2. qRT-PCR analysis was performed by following three biological replicates per sample, as reported earlier [54]. The relative expression levels of putative identified CmGGP1 and associated genes were determined using an earlier reported method [55], respectively.

### 2.5. Statistical Data Analysis

The experiment dataset was recorded in numerical values using Microsoft Excel Sheet (version 2021). The final arranged data were analyzed and visualized using GraphPad Prism software (version 9.0) and statistical analysis was performed at probability levels of *p* < 0.01 and *p* < 0.05, respectively.

## 3. Results

### 3.1. Analysis of Identified CmGGP1 Gene in Melon Genome

The preliminary identification results of the GGP gene were obtained from the reference genome of melon_DHL92_V4. The gene density across the whole genome chromosomes was checked and filtered, which showed a single genetic locus between a 12,223,955 and 12,227,154 base pair (bp) interval over chromosome 1 (Chr01), containing only one target gene (MELO3C013136.2) named CmGGP1 (Figure 1A). The gene structure contains 5′ and 3′ UTRs, seven exons, and six introns; the full length of the gene was 3200 bp and the coding region was 1359 bp, encoding a total of ~452 amino acids (aa) (Figure 1B). The secondary structure characteristics of the CmGGP1 protein seemed to be composed of a random coil (44.69%), followed by an alpha (α) helix (33.41%), and an extended strand (17.04%), respectively (Figure 1C).

The CmGGP1 protein model was predicted using the AlphaFold v2 database and the average pLDDT model confidence was 81.44, consistent with the melon A0A1S3BIN8 (A0A1S3BIN8_CUCME) model in the SWISS-MODEL database (Figure 1D). The plant-mPLoc subcell prediction exhibited that the CmGGP1 protein was localized in the chloroplast or nucleus, and the protein prediction results of SignalP (version 5.0) showed that the CmGGP1 protein did not have a signal peptide (Supplementary Figure S1). The TMHMM tool (version 2.0) analysis predicted that the CmGGP1 protein has no transmembrane structure either inside or outside (Supplementary Figure S2).

### 3.2. Analysis of CmGGP1 Gene Evolutionary Relationship

**Figure 2:**
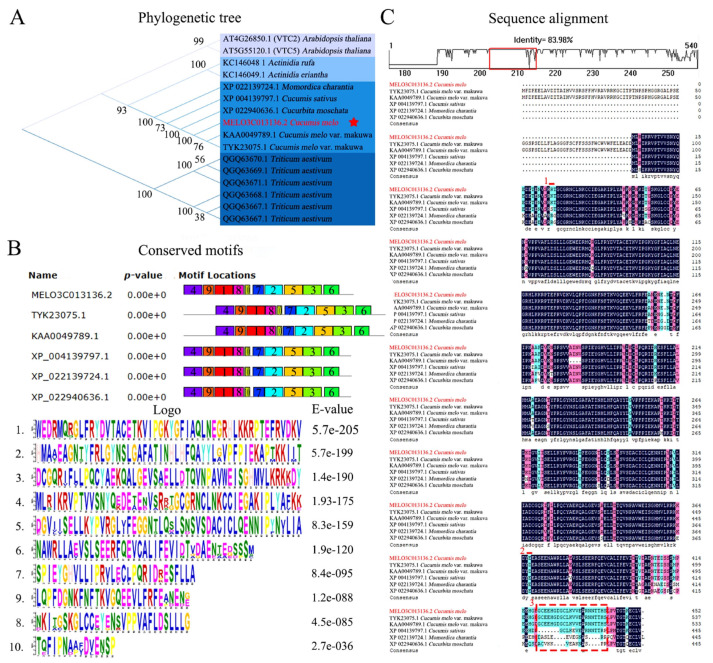
Phylogenetic and structural variation analysis of the CmGGP1 protein in Cucurbitaceae family. (**A**) Analysis of evolutionary relationship differences in multi-species. The red star indicate the conserved evolution of melon. (**B**) Analysis of conserved motifs. (**C**) Analysis of pairwise sequence alignment in the Cucurbitaceae family.

According to the phylogenetic tree analysis, it was noticed that the *CmGGP1* gene in melon evolution is very conservative. The multi-species evolutionary relationships are mainly divided into four family categories, including Cruciferae, Kiwifruit, Cucurbitaceae, and Graminaceae (Figure 2A). The Cucurbitaceae family contains five species, as follows: Bitter gourd (*Momordica charantia*), Cucumber (*Cucumis sativus*), Squash (*Cucurbita moschata*), Melon (*Cucumis melo*), and Oriental melon (*Cucumis melo* var. Makuva), which exhibited highly consistent motif elements in six GGP protein sequences (MELO3C013136.2, XP_022139724.1, XP_004139797.1, XP_022940636.1, TYK23075.1, and KAA0049789.1). The structural analysis of six proteins of Cucurbitaceae was also conducted, which showed that the ten main motif elements were completely consistent and the similarity of six proteins was exhibited to be highly conserved (Figure 2B).

The multiple sequence analysis of six proteins of the Cucurbitaceae family showed a differentiated structure, and a high sequence similarity index (83.98%) was observed (Figure 2C). We found a highly variable region at the 3′ end of the protein that can be used to distinguish the Cucurbitaceae species, and melon (*Cucumis melo*) and oriental melon (*Cucumis melo* var., Makuva) are indistinguishable in this interval. Two GGP protein sequences (TYK23075.1 and KAA0049789.1) in *Cucumis melo* var., Makuva showed the most significant structural variation compared with other cucurbit species; however, the GGP protein (TYK23075.1) has four amino acids inserted relative to the KAA0049789.1 protein sequence, which may lead to the differentiation in gene function. The functional prediction of cucurbit family proteins also showed that the six GGP proteins belong to the DUF4922 superfamily (Supplementary Figure S3), whose functional domain has not been fully annotated in other studies. Hence, we assumed that this is currently identified as a new protein and classified as having GDP-D-glucose phosphorylase 1 family domain architecture ID 52482. These are kinds of very specific and efficient enzymes that have the main function of regulating the level of GDP-D-glucose in cells.

### 3.3. Analysis of CmGGP1 Protein in Melon Genome

**Figure 3:**
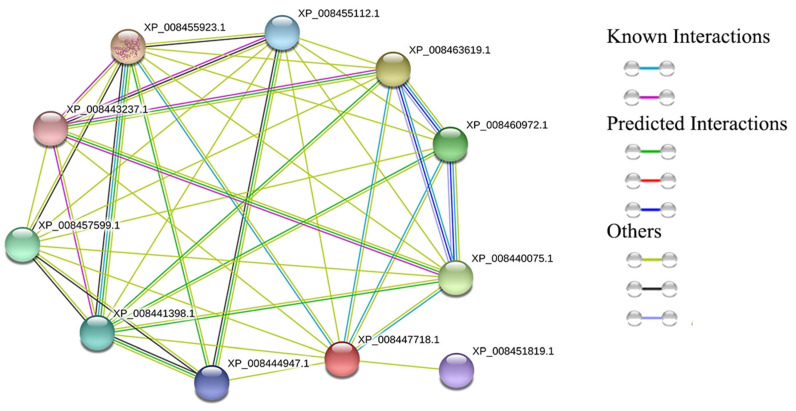
Analysis of CmGGP1 protein interaction network in melon genome.

According to the protein analysis, the XP_008447718.1 (MELO3C013136.2.1) protein was exhibited as a candidate protein encoding the GDP-L-galactose phosphorylase 1 (CmGGP1) pathway in melon, as shown in Table 1 and Table 2, and Supplementary Table S3. The CmGGP1 protein (MELO3C013136.2.1) interaction network analysis in melon showed 11 nodes, 41 edges, PPI enrichment *p*-value < 5.97 × 10^−13^, average node degree (7.45), and average local clustering coefficient (0.915) (Figure 3 and Supplementary Table S3). The CmGGP1 protein (MELO3C013136.2.1) in the Cucurbit database was highly consistent with the melon protein (XP_008447718.1) (Figure 3). There were mainly 10 proteins interacting with the CmGGP1 protein and seven proteins (XP_008440075.1, XP_008447718.1, XP_008455112.1, XP_008455923.1, XP_008457599.1, XP_008460972.1, and XP_008463619.1) seemed to be involved in the ascorbate and aldarate metabolism pathway (cmo00053) (Table 1 and Table 2, and Supplementary Table S3).

Among these seven proteins, a total of four proteins (XP_008463619.1, XP_008460972.1, XP_008455923.1, and XP_008440075.1) were predicted with the interaction threshold value scores of >0.95, and three other proteins (XP_008457599.1, XP_00845512.1, and XP_008451819.1) had interaction threshold values of >0.7 with the CmGGP1 protein (Table 3 and Supplementary Table S3). These might have a main function in the ascorbate and aldarate metabolism pathway, but its interaction relationship with the melon CmGGP1 protein has not been reported at present. However, it has been preliminarily identified in other species as co-regulating AsA synthesis under drought stress and light treatment, and we also focused on these identified proteins.

### 3.4. Analysis of Comparative Genomic Characterization of CmGGP1 Gene within Different Germplasms of Melon

**Figure 4:**
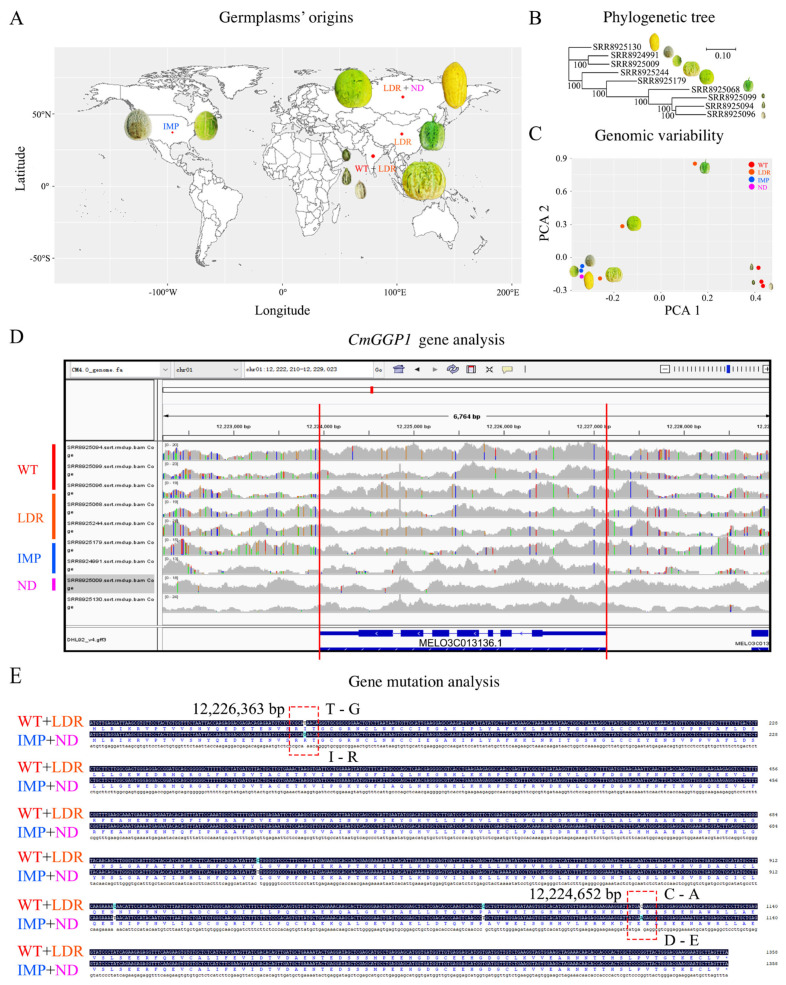
CmGGP1 gene characterization in the genome of four melon germplasms. (**A**) Geographical origins of germplasms. (**B**) Genomic evolutionary relationships analysis. (**C**) PCA analysis for genomic variability. (**D**) *CmGGP1* gene analysis based on comparative whole genome resequencing. (**E**) Comparative analysis of *CmGGP1* gene sequence mutation, respectively. WT, wild-type; LDR, landrace; IMP, improved-type; ND, not defined. The area marked with red dotted boxes denotes the candidate gene mutation sites among the comparative genomes of four melon germplasms.

The worldwide map view of WT, LDR, IMP, and ND germplasm resources belonging to different geographical origins showed that WT, LDR, and IMP were classified as domesticated materials, but ND germplasms are not known from the domestication type and come from Russia (Figure 4A). The constructed phylogenetic tree depicted the significant genomic evolutionary relationships in different clades among the four melon germplasm resources (Figure 4B).

Further, the principal component analysis (PCA) plot exposed the major variability along two axes (PC1 and PC2), which depicted that these four germplasm resources are well separated from each other and consistent with their geographical origins. The varieties of WT and IMP germplasm seemed concentrated and showed obvious differences. A highly variable genomic variation was observed in local varieties; however, ND genome showed a more close resemblance with IMP material (Figure 4C). Regarding the *CmGGP1* gene (MELO3C013136.2), there were evolutionary differences in the gene structure among the melon germplasm materials. The *CmGGP1* gene structure in WT and LDR germplasm materials exhibited a relatively close association and abundant structural variations were observed as compared to IMP and ND germplasm materials (Figure 4D).

A total of five mutation sites were mainly identified in the candidate gene-coding region of comparative genome sequences of four germplasm resources, but two of them had non-synonymous mutations that similarly altered the amino acid sequences in two melon varieties “Cinderella melon (LDR-type) and PMR45 (IMP-type)”. The A–G base mutation (changing adenine to guanine) occurred at about 12,226,363 bp, which changed the amino acids from K to R (lycine (lys) to arginine (Arg)). The other C–A base mutation (cytosine to adenine) was observed at 12,224,652 bp that changed the amino acids from D to E (aspartate (Asp) to glutamic acid (Glu)). These non-synonymous mutations were effectively observed among the comparative genome sequences of WT versus LDR, IMP versus ND germplasm materials, and the reference genome of melon_DHL92_V4 (Figure 4E and Supplementary Table S4), exhibiting evolutionary differences during the domestication process. Thus, our analysis strongly suggests that the *CmGGP1* gene may be affected by artificial breeding and the AsA synthesis function has changed in the melon germplasm resources of two subspecies.

### 3.5. Transcriptome Analysis of CmGGP1 and Interacting Genes in Different Tissues of Melon

**Figure 5:**
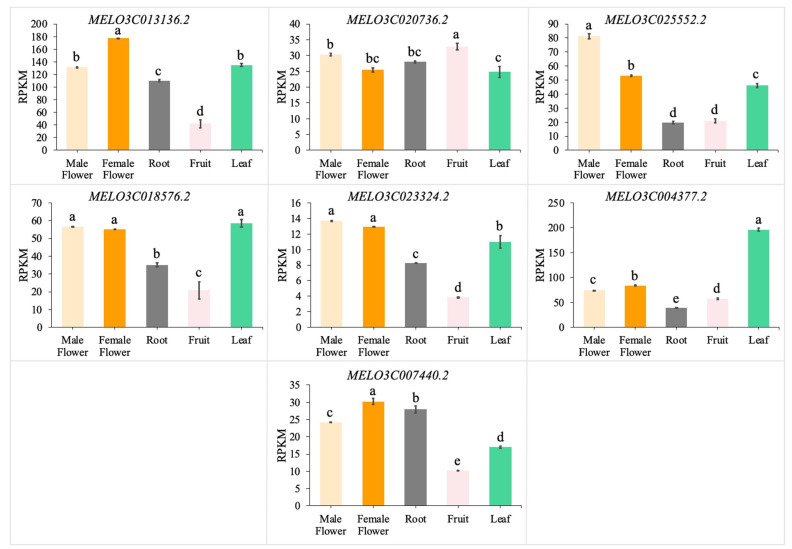
Tissue specificity expression analysis of identified *CmGGP1* gene and its putative interactive genes in melon. RPKM—reads per kilobase million. The statistical letters (a–e) indicate that the significant differences were observed at a probability level of *p* < 0.05.

The *CmGGP1* gene and its associated interactive genes were analyzed in different tissues (male flower, female flower, root, fruit, and leaf) of melon plants using transcriptome sequencing. The tissue expression specificity analysis of seven identified putative genes (MELO3C013136.2, MELO3C020736.2, MELO3C025552.2, MELO3C018576.2, MELO3C023324.2, MELO3C0004377.2, and MELO3C007440.2) involved in ascorbate and aldarate metabolism pathways were checked (Figure 5). The results showed that the *CmGGP1* gene (MELO3C013136.2) and its interactive gene expression were highly expressed in female flowers, male flowers, and leaves, respectively. However, it is observable that the other six genes have relatively lower expression in the roots and fruits, except for the MELO3C020736.2 gene. Therefore, it is speculated that the *CmGGP1* gene demonstrated higher levels of expression, modulating the AsA biosynthesis pathway in flowers and leaves more so than that in roots and fruits, respectively.

### 3.6. Analysis of AsA Content in Melon Leaves and CmGGP1 Gene Expression Pattern

**Figure 6:**
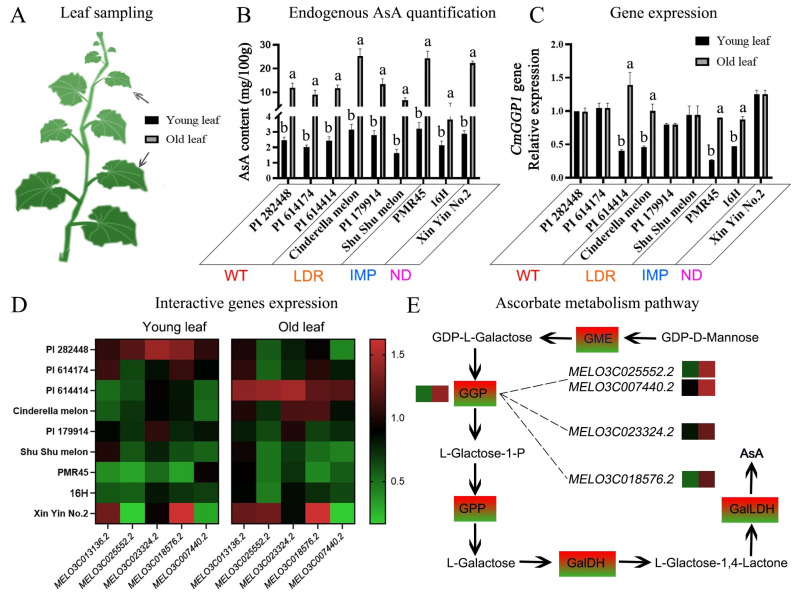
Endogenous quantification of AsA content and expression pattern analysis in the young and old leaves of differentiated melon varieties. (**A**) Leaf sampling diagram. (**B**) Endogenous AsA content levels. (**C**) CmGGP1 gene expression patterns. (**D**) Heat maps of expression patterns of the CmGGP1 gene and related interactive genes. (**E**) Genes modulating the AsA biosynthesis pathway model. The statistical letters (a and b) indicate that the significant differences were observed at a probability level of *p* < 0.01.

The young and old leaves were sampled from different varieties of domesticated melon germplasm, as shown in the model diagram (Figure 6A). The endogenous synthesis of AsA content (mg/100 g) showed an obvious accumulation effect and significant differences for AsA synthesis and metabolism; however, the AsA content in young leaves was significantly lower, as compared to the old leaves, for all melon materials (Figure 6B).

The qRT-PCR analysis of *CmGGP1* gene expression patterns showed that the expression level of four melon varieties (PI 614414, Cinderella melon, PMR45, and 16H) was significantly increased in the old leaves than that of young leaves (Figure 6C), while the gene expression level of five melon varieties (PI 282448, PI 614174, PI179914, Shu Shu melon, and Xin Yin No.2) remained stable in both young and old leaves, having no visualized significant differences, respectively. Interestingly, we found that the expression of the *CmGGP1* gene (an important rate-limiting enzyme in AsA synthesis) was stable in the young and old leaves of five materials, while the content of AsA was increased in all these materials. This indicated that AsA was continuously synthesized during the development of leaves and that AsA metabolism was not greater than AsA biosynthesis.

Further, the *CmGGP1* gene and interactive gene expression patterns exhibited that two Inositol-1-monophosphatase linked genes (*MELO3C025552.2* and *MELO3C007440.2*), one bifunctional phosphatase IMPL2 related gene (*MELO3C02332.2*), and one GDP-mannose 3,5-epimerase 2 isoform X1 related gene (*MELO3C018576.2*) were significantly upsurged in most of the melon varieties during leaf development and that their gene expression patterns were similar to those of *CmGGP1* expression in young and old leaves (Figure 6D). For the known L-galactose pathway to synthesize the AsA pathway genes, we can see that the interactive expressions of four candidate genes (*MELO3C025552.2*, *MELO3C007440.2*, *MELO3C023324.2*, and *MELO3C018576.2*) had similar patterns to the *CmGGP1* gene expression in the young and old leaves of the melon variety (PI 614414), depicting the regulatory mechanism of the ascorbate metabolism pathway (Figure 6E). Overall, the experimental results and analysis exhibited that these four genes might interact with the *CmGGP1* gene and modulate the AsA biosynthesis in melon plant leaves.

## 4. Discussion

### 4.1. There Is Only One Gene (CmGGP1) Contributing to the L-Galactose Pathway, Modulating AsA Biosynthesis in Melon Plants

In the former study of Tao et al. [56], it was found that among the 71 plant species, 50 species contain two or more copies of GGP genes, which are mainly distributed in the lineage of angiosperms and gymnosperms, while the species containing only one copy of the *GGP* gene are found mainly in the lineage of chlorophytes, and only 10 of the 41 dicotyledonous species have a single *GGP* gene [56]. Melons are dicotyledonous plants and there is also the presence of one single *GGP* gene, as mentioned in the above-stated plant groups, but this case is very rare. According to our genome-wide bioinformatics analysis, we also identified only one gene (*CmGGP1*) in the improved reference genome of melon_DHL92_V4, located on the Chr01 segment (Figure 1), depicting a consistent but extremely rare result. It is supposed that this may possibly be related to the occurrence of whole genome duplication (WGD) events, while it has been stated in previous studies that no WGD events occurred in the whole genome studies of melon [57] and cucumber [58].

Moreover, it has been reported that the evolution of the plant *GGP* gene family is primarily restricted by purification selection, indicating the functional significance and conservation of the *GGP* gene in its evolutionary progression [56]. Therefore, the *CmGGP1* gene is particularly known as a key rate-limiting enzyme gene involved in the L-galactose pathway controlling AsA synthesis in melon. It was proposed that the CmGGP1 protein of melon is predicted to have no transmembrane domain, which is consistent with the protein structure of the *VTC2* and *VTC5* genes in *Arabidopsis thaliana* [20]. Herein, our subcellular localization analysis similarly predicted that the CmGGP1 protein was located in the chloroplast or nucleus (Supplementary Figures S1 and S2). Although it has not been confirmed in melon, its homologous protein has been confirmed in *Arabidopsis thaliana*. GGP protein in *Arabidopsis thaliana* was identified intracellularly by using GFP-labeled protein and there was also a fluorescent signal in the nucleus [59], indicating that the *GGP* gene not only has a significant role in the intracellular network, but also has a certain nuclear function. However, it is generally believed that AsA synthesis occurs mainly in the cytoplasm, including GMP [60], GME [18], GGP [21], GPP [22,28], and GalDH [26], and the six key enzymes forming L-Glactose 1, 4-Lactone are oxidized to AsA by the L-GalLDH when they cross the outer membrane of mitochondria [61]. Our comprehensive bioinformatics analysis revealed only one GGP protein encoded by the *CmGGP1* gene in melon, which may be an important enzyme involved in the L-galactose pathway controlling AsA biosynthesis in the cytoplasm. However, the GGP protein function in the nucleus still needs further study at an in-depth level.

### 4.2. GGP Gene Family Evolution Is Very Conserved and Protein Structure Has Highly Conserved Characteristics

Regarding the analysis of phylogenetic association, we used earlier published protein sequences from multiple plant species and found that they could be categorized into four types of different crop families, as follows: Cucurbitaceae, Cruciferae, Actinidiaceae, and Lepidaceae. The GGP proteins from the Cucurbitaceae family depicted highly similar sequence structures and conserved motifs. The similarity index of six protein sequences from different species of the Cucurbitaceae family was 83.98%, and 10 motif sequences were highly similar (Figure 2). However, the high consistency of GGP protein sequences in the Cucurbitaceae family suggested that similar catalytic functions may exist.

In earlier studies, genome-wide analysis identified a total of six *GGP* genes in bread wheat, except for *TaGGP2-D*, which could not be differentiated due to Agrobacterium technology [36], but the AsA content of *Arabidopsis thaliana* was significantly upsurged in varying degrees after the instantaneous transformation of the remaining five *GGP* genes. However, subtle differences in homologous genes may also lead to functional differentiation, e.g., two genes (*VTC2* and *VTC5*) encoding GGP were spotted in *Arabidopsis thaliana*, but only the expression trend of the *VTC2* gene showed significant expression affecting the AsA biosynthesis [21]. It was shown that the CSN5B engages with *VTC1*, influencing the modulation of AsA biosynthesis in *Arabidopsis thaliana* [62]. The earlier study findings of *GGP* genes showed that both *SlGGP1* and *SlGGP2* genes were expressed in tomato fruits, but *SlGGP1* primarily showed contribution for the regulation of AsA content during fruit development, while *SlGGP2* is associated with the fruit ripening process [4]; but, these two homologous genes showed some functional differentiation. 

Herein, we also found two highly homologous GGP proteins in *Cucumis melo* var. makuva, which may have a similar phenomenon (Figure 3). However, only the *CmGGP1* gene encoding GGP exists in melon, belonging to the Cucurbitaceae family, so the biosynthesis of AsA in different tissues of melon may be regulated by the *CmGGP1* gene, and this gene plays a vital role in the metabolism pathway of AsA synthesis in melon. In addition, a comparative whole genome analysis of the *CmGGP1* gene from different domesticated melon germplasms exhibited that WT and LDR germplasms were more similar, and the ND was observed closer to IMP germplasm. A total of two non-synonymous type mutations between the *CmGGP1* gene of these two materials were also identified (Figure 4), which perhaps indicated that the function of this gene may be changed. This means that the AsA biosynthesis of melon may have changed during domestication.

### 4.3. CmGGP1 Gene Has a Tissue Specificity Expression Conferring AsA Biosynthesis in Melon

In earlier studies, it has been stated that light is an important factor affecting AsA biosynthesis, and female flowers, male flowers, and leaves are important tissues for receiving sunlight signals [38,39]. A total of four proteins (XP_008463619.1, XP_008460972.1, XP_008455923.1, and XP_008440075.1) have been focused on, although their interaction relationship with the melon CmGGP1 protein has not been reported at present. However, it has been preliminarily identified in other species as co-regulating AsA synthesis under drought stress and light treatment [1,63,64].

Herein, we identified that the interaction threshold value scores predicted by the STRING platform were all greater than 0.95 (Table 3). We also checked *CmGGP1* gene expression through different tissue expression specificity analysis of the flowers, leaves, fruits, and roots of melon and results revealed that *CmGGP1* gene expression in flowers and leaves was significantly higher than that in roots and fruits (Figure 5). This result was in accordance with the previous findings of *Arabidopsis thaliana* studies, showing the *VTC2* and *VTC5* expression in roots, stems, leaves, and flowers [20,59]. GGP is considered to be an important rate-limiting enzyme in the process of AsA biosynthesis [28]. In melon plants, the flowers and leaves are the main organs that receive sunlight signals as compared with fruits and roots, and light affects AsA biosynthesis. As an antioxidant molecule, the AsA product is widely present in various tissues of plants and essentially contributes to photosynthesis, plant cell wall formation, fruit softening and aging, enhancing plant stress resistance, etc. [1,65,66]. These findings imply that *CmGGP1* may play a significant function in various tissues of melon. Previous studies have similarly shown that *GGP* gene expression and AsA biosynthesis are significantly increased when exposed to light for a long time [20,67]. For example, in tomatoes, the effect of light treatment on the AsA content change in leaves was greater than that in fruits [68]. Moreover, it was found that the AsA content in fruits was not affected by the biosynthesis of AsA content in leaves, which may indicate that AsA does not exist in inter-tissue transport and accumulation [69] and is only synthesized in cells of different tissues to participate in the physiological development process of plants.

Herein, we analyzed the endogenous synthesis of AsA content in nine melon varieties of different germplasms during plant growth. It was found that the AsA content in old leaves was generally higher compared to young leaves. Further, the relative expression of the *CmGGP1* gene in old leaves was also higher than that in young leaves; however, no substantial differentiation in *CmGGP1* gene expression was observed in five melon materials, while AsA was increased in old leaves (Figure 6). Some earlier studies also depicted that the dynamic change of AsA content in plants during development has a certain relationship with species characteristics, e.g., ascorbic acid increases with fruit ripening in tomatoes, grapes, citrus, and strawberries during each development and growth stage [7,34,46,66,69,70], which is also consistent with our results of AsA synthesis in melon. However, AsA content in peach fruits decreased with fruit ripening, which was inconsistent with our results [71]. AsA content in kiwifruit was high in the immature stage but decreased with fruit maturity and finally stabilized at a certain concentration until full maturity [12,70], which was inconsistent with our results. Whether the AsA dynamic pattern in melon fruits is consistent with that in leaves needs further study.

### 4.4. The Co-Expressed Genes Indicated the Possible Interaction with the CmGGP1 Gene

We performed the protein interaction network prediction in melon and found that six proteins (like the L-galactose and the inositol pathway) may interact with the CmGGP1 protein (Figure 3); however, two of the proteins “MELO3C018576.2.1 (L-galactose dehydrogenase) and MELO3C020736.2.1 (L-Galactono-1,4-lactone)” were also identified for significant interaction (Table 2). Previous studies also exposed that dehydrogenase is directly involved in the biosynthesis of AsA by the L-galactose pathway. GPP converts L-galactose 1-phosphate to L-galactose [22,23]. L-Galactono-1,4-lactone dehydrogenase (GalLDH) changes L-Glactose 1,4-Lactone into AsA [25,26,72]. Therefore, it is supposed that MELO3C018576.2.1 and MELO3C020736.2.1 proteins may be directly involved in the AsA biosynthesis in melon plant. Thus, we also focused on the analysis of the other four proteins, e.g., MELO3C025552.2.1 and MELO3C007440.2.1, both of which are inositol-1-monophosphatases, MELO3C023324.2.1 function annotation is bifunctional phosphatase IMPL2, and MELO3C018576.2.1 function annotation is GDP-mannose 3,5-Epimerase 2 isoform X1.

It was found that the Inositol-1-monophosphatase (IMP) is an indispensable enzyme in the Inositol-1-monophosphatase metabolic pathway, which has the function of dephosphorylating inositol-1-monophosphatase and participates in multiple metabolic and signaling pathways in chickpeas (*Cicer arietinum* L.) and the improved-type germplasm not only participated in inositol biosynthesis, but also depicts the overexpression of CalIMP that significantly affected AsA biosynthesis [73,74]. However, it was observed that MELO3C025552.2.1 and MELO3C007440.2.1 have similar functions and need further study for strong validation. The earlier studies have shown that improved-type germplasm material has a hydrolytic L-galactose 1-phosphate (L-Gal 1-P), which is a substrate for GGP [22], suggesting that IMP may affect endogenous AsA biosynthesis by affecting the concentration of the GGP substrate. The IMPL2 participates in the histidine synthesis process [75], but not in the hydrolysis of inositol and galactose phosphate in plant cells [76]. It was supposed that MELO3C023324.2.1 protein may not participate in the synthesis of AsA in melon and its specific functions need to be further explored. GDP-mannose 3,5 epimerase and VTC2 are well known as key hubs for the synthesis of GDP-hexoses and L-galactose 1-phosphate [28,77], further extending the VTC2 cycle, which connects photosynthesis activity with AsA biosynthesis and plant cell wall metabolism.

Herein, we found that two genes (*MELO3C025552.2* and *MELO3C007440.2*) of Inositol-1-monophosphatase, one gene (*MELO3C02332.2*) of bifunctional phosphatase IMPL2, and one gene (*MELO3C018577.2*) of GDP-mannose 3,5-epimerase 2 isoform X1, were significantly upsurged in melon materials during leaf development, and its gene expression patterns were similar to those of the *CmGGP1* gene in young and old leaves (Figure 6). In short, we speculated that the MELO3C018576.2.1 protein may have a direct interaction with the *CmGGP1* gene to affect AsA biosynthesis in melons.

## 5. Conclusions

In this study, we identified a single AsA synthesis rate-limiting enzyme gene (*CmGGP1*) in the melon genome using bioinformatics analysis and found that the CmGGP1 protein has its main functions in the chloroplast or nucleus. The multiple sequence alignment analysis showed that the CmGGP1 protein has a highly conserved motif in cucurbit crops and suggested that the *CmGGP1* gene was selected by domestication during evolution. Although the gene was different in different domestication materials, AsA biosynthesis was not greatly affected during plant development. The internal AsA quantification and interactive gene expression analysis in melon leaves showed that AsA biosynthesis in leaves was greater than AsA metabolic consumption, and four possible interactive genes linked with *CmGGP1* were revealed in the ascorbate metabolism pathway. In short, our research findings deliver a theoretical basis for an in-depth study of the AsA biosynthesis pathway in melon, which will further help in developing improved cultivars based on marker-assisted selection and breeding approaches.

## Figures and Tables

**Table 1:** Analysis of metabolic pathways of interacting proteins.

Pathway	Description	Gene Set Counts	*p*-Value
cmo00053	Ascorbate and aldarate metabolism	7 of 45	2.35 × 10^−15^
cmo01110	Biosynthesis of secondary metabolites	9 of 958	4.84 × 10^−10^
cmo01100	Metabolic pathways	7 of 1685	4.75 × 10^−8^
cmo04070	Phosphatidylinositol signaling system	3 of 53	1.12 × 10^−5^
cmo00562	Inositol phosphate metabolism	3 of 58	1.16 × 10^−5^
cmo00520	Amino sugar and nucleotide sugar metabolism	3 of 114	6.87 × 10^−5^

**Table 2:** Annotation information of ascorbate and aldarate metabolism protein.

NCBI Database	CuGenDB	Annotation
XP_008447718.1	MELO3C013136.2.1	GDP-L-galactose phosphorylase 1
XP_008457599.1	MELO3C020736.2.1	L-galactono-1,4-lactone dehydrogenase, mitochondrial
XP_008463619.1	MELO3C025552.2.1	Inositol-1-monophosphatase
XP_008455112.1	MELO3C018576.2.1	L-galactose dehydrogenase
XP_008460972.1	MELO3C023324.2.1	Bifunctional phosphatase IMPL2, chloroplastic isoform X1
XP_008455923.1	MELO3C018576.2.1	GDP-mannose 3,5-epimerase 2 isoform X1
XP_008440075.1	MELO3C007440.2.1	Inositol-1-monophosphatase; Inositol-phosphate phosphatase-like

**Table 3:** Protein interaction prediction analysis.

Node1	Node2	Score
XP_008447718.1	XP_008455923.1	0.984
XP_008447718.1	XP_008440075.1	0.970
XP_008447718.1	XP_008463619.1	0.970
XP_008447718.1	XP_008460972.1	0.967
XP_008447718.1	XP_008457599.1	0.853
XP_008447718.1	XP_008455112.1	0.811
XP_008447718.1	XP_008451819.1	0.708

## Data Availability

Available upon reasonable request to the corresponding authors.

## Supplementary Materials

The following supporting information can be downloaded at: https://www.mdpi.com/article/10.3390/antiox13040397/s1, Figure S1: Prediction results of CmGGP1 protein type and signal peptide; Figure S2: Prediction results of CmGGP1 protein transmembrane structure; Figure S3: Comparative functional prediction analysis; Table S1: Detailed information of four types of melon germplasm used in this study; Table S2: The information of all gene primers used in this study; Table S3: Analysis of the CmGGP1 protein interaction network; Table S4: *CmGGP1* gene mutation difference in detected SNP loci among four types of melon germplasm resources based on whole genome resequencing.
