# Supplementary figures and images for: Deciphering the Genomic Characterization of the GGP Gene Family and Expression Verification of CmGGP1 Modulating Ascorbic Acid Biosynthesis in Melon Plants
Source: Antioxidants (Basel). 2024 Mar 26;13(4):397. doi: 10.3390/antiox13040397 (PMC11047344; doi:10.3390/antiox13040397)

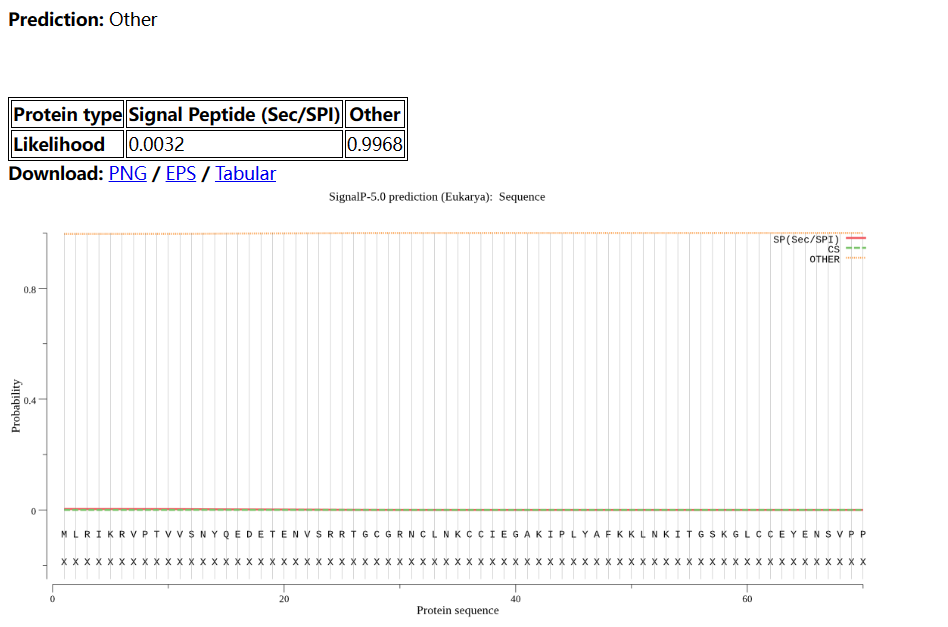


**Supplementary Figure S1:** Prediction results of CmGGP1 protein type and signal peptide.

Supplement: Supplementary file 1 [file antioxidants-13-00397-s001.zip › Supplementary Material/Supplementary Figure S1.docx]

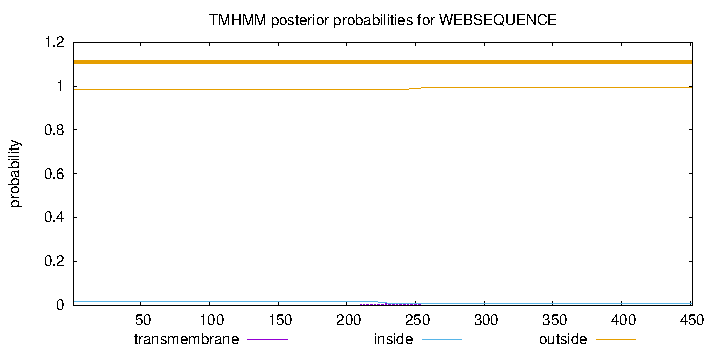


**Supplementary Figure S2:** Prediction results of CmGGP1 protein transmembrane structure.

Supplement: Supplementary file 1 [file antioxidants-13-00397-s001.zip › Supplementary Material/Supplementary Figure S2.docx]

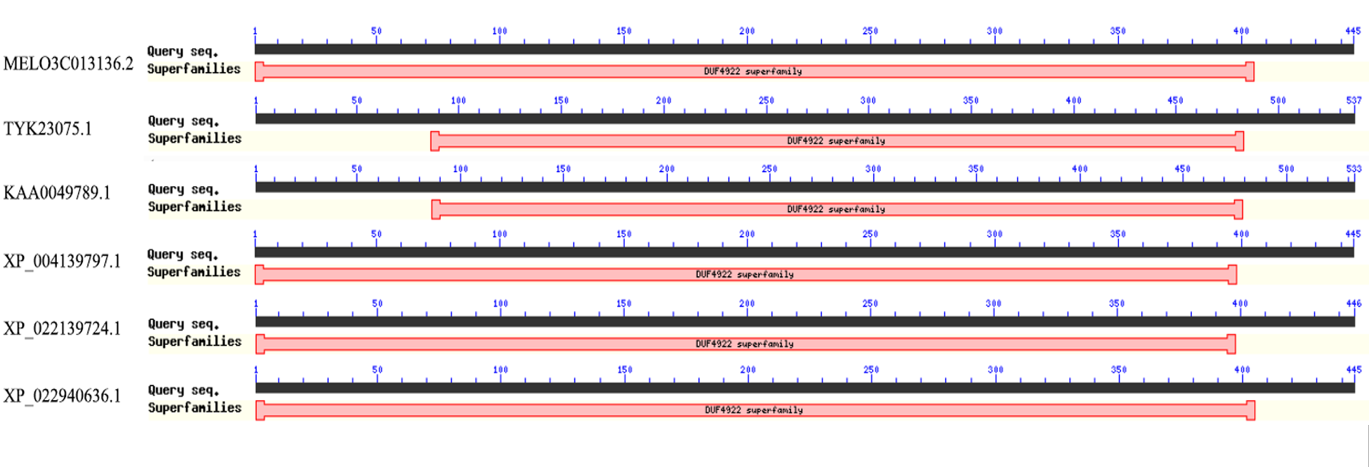


**Supplementary Figure S3:** Comparative functional prediction analysis.

Supplement: Supplementary file 1 [file antioxidants-13-00397-s001.zip › Supplementary Material/Supplementary Figure S3.docx]
